# Modulation of the rhizosphere microbiome structure and optimization of beneficial functions in winter wheat induced by *Bacillus subtilis*: a metagenomic and phenotypic study

**DOI:** 10.1093/femsec/fiaf097

**Published:** 2025-09-30

**Authors:** Mykola Patyka, Renjun Wang, Anastasiia Honchar, Tetiana Patyka, Serhii Khablak

**Affiliations:** Faculty of Life Sciences, Qufu Normal University, No 57 West Road, Jingxuan, Qufu 273165, China; National University of Life and Environmental Sciences of Ukraine, 15 Heroiv Oborony Str., Kyiv 03041, Ukraine; Faculty of Life Sciences, Qufu Normal University, No 57 West Road, Jingxuan, Qufu 273165, China; National University of Life and Environmental Sciences of Ukraine, 15 Heroiv Oborony Str., Kyiv 03041, Ukraine; Faculty of Life Sciences, Qufu Normal University, No 57 West Road, Jingxuan, Qufu 273165, China; National University of Life and Environmental Sciences of Ukraine, 15 Heroiv Oborony Str., Kyiv 03041, Ukraine; Institute of Food Biotechnology and Genomics of the National Academy of Sciences of Ukraine, 2A Baida-Vyshnevetsky Str., Kyiv 04123, Ukraine

**Keywords:** 16S rRNA, *Bacillus subtilis*, metagenome, microbiome, plant growth promotion, plant–microbe interactions, rhizosphere, shotgun metagenomics, soil health, *Triticum aestivum*

## Abstract

The rhizosphere microbiome critically determines plant health and productivity. This study investigated the impact of *Bacillus subtilis* H38 on the taxonomic and functional profiles of the winter wheat (*Triticum aestivum* L.) rhizosphere microbiome under typical chernozem conditions using 16S rRNA gene sequencing and shotgun metagenomics, complemented by plant phenotypic evaluation and targeted metabolite analysis. Inoculation with *B. subtilis* H38 significantly restructured the rhizosphere bacterial community, increasing alpha-diversity (Shannon index from 5.8 to 6.7) and showing distinct clustering in beta-diversity analysis. The relative abundance of putative plant-beneficial genera, including *Bacillus, Pseudomonas, Azotobacter*, and *Streptomyces*, was significantly elevated. Shotgun metagenomic analysis revealed enrichment of functional genes associated with nitrogen fixation, phosphorus mobilization, phytohormone biosynthesis, siderophore production, and synthesis of antimicrobial compounds. Targeted metabolomic analysis confirmed elevated levels of indole-3-acetic acid (IAA) and key siderophores. Concurrently, treated wheat plants exhibited an 18.0% increase in aboveground biomass and a 25.0% increase in root length under field conditions. These findings underscore the potential of *B. subtilis* to beneficially reshape the rhizosphere microbiome and its metagenome, leading to enhanced plant growth, and highlight its utility as a potent biofertilizer for improving wheat productivity. This research reinforces the potential of harnessing beneficial plant–microbe interactions to enhance agricultural productivity while minimizing dependence on synthetic agrochemicals.

## Teaser

Our research demonstrates how beneficial *Bacillus subtilis* bacteria reshape soil microbes, significantly enhancing wheat growth and offering an ecofriendly solution for sustainable agriculture.

## Introduction

The rhizosphere, the narrow zone of soil directly influenced by plant roots, is a dynamic ecosystem where intensive plant–microbe interactions critically determine plant nutrition, protection against pathogens, and adaptation to environmental stressors. The composition and functional capabilities of these microbial communities are shaped by a variety of factors, including soil type, plant genotype, and agronomic practices (Berendsen et al. 2012, Philippot et al. 2013, Qu et al. 2020). Conventional agriculture often relies on synthetic agrochemicals, which can have detrimental environmental effects (Gnanaprakasam and Vanisree 2022, Dhuldhaj et al. 2023, Ahmad et al. 2024). Biofertilizers based on soil microorganisms, particularly plant growth-promoting rhizobacteria (PGPR), represent an environmentally friendly alternative to enhance crop yields and improve soil health. The genus *Bacillus*, and especially *Bacillus subtilis*, is well-known for its multifaceted plant-beneficial properties, including nutrient solubilization and synthesis of a broad spectrum of antimicrobial compounds (Garbeva et al. 2004, Gopalakrishnan et al. 2015, Honchar et al. 2021a). While many studies have documented the effects of specific *Bacillus* strains on plant growth, a comprehensive understanding of their impact on the holistic taxonomic structure remains an important area of research. This study, therefore, focuses on *B. subtilis* H38, a strain selected from preliminary *in vitro* and greenhouse trials for its proven high plant-growth-promoting and antagonistic activity against major phytopathogens. (Ongena and Jacques 2008, Honchar et al. 2021b, Beghini et al. 2021). Modern high-throughput sequencing technologies, such as 16S rRNA gene amplicon sequencing and shotgun metagenomics, offer powerful tools to characterize the diversity and functional potential of these complex microbial communities (Quince et al. 2017, Imchen et al. 2019, Frey et al. 2022, Shami et al. 2022, Jiang et al. 2024). Therefore, the primary objective of this study was to comprehensively assess the impact of *B. subtilis* H38 on the taxonomic composition and functional metagenomic profile of the winter wheat rhizosphere microbiome. We hypothesized that the application of *B. subtilis* H38 would significantly alter the structure of the microbial community, enriching it with beneficial taxa and functional genes associated with plant growth promotion and health, which would consequently lead to measurable improvements in plant growth parameters.

## Materials and methods

### Experimental design and sample collection

The soil type was a typical Chernozem with the following properties in the arable layer: humus content of 4.2%–4.4%, plant-available phosphorus (P_2_O_5_) of 45–55 mg/kg, exchangeable potassium (K_2_O) of 150–160 mg/kg, readily hydrolyzable nitrogen of 124–140 mg/kg, and a salt extract pH of 6.9.

The model crop was winter wheat (*Triticum aestivum* L.), which is characterized by ecological diversity and resistance to phytopathogens. The optimal germination temperatures of the crop are: minimum, 1°C–20°C; favorable, 12°C–18°C; optimal, 24°C–28°C; and maximum, 36°C–38°C. The experiment was established in a randomized complete block design (RCBD) with two treatments and five replicates for each treatment.

The experiment was established in a RCBD with two treatments and five replicates for each treatment. The individual plot size was 12 m^2^. The experimental treatments were:

- Control (C): seeds were sown without treatment with *the B. subtilis* H38 strain, plants were sprayed with water.
- Biological treatment (BS): Seeds were treated before sowing with a suspension of the *B. subtilis* H38 strain (titer: 1.8 × 10^9^ CFU/ml) at a rate of 1 l/t. This was followed by a foliar application of the same bioagent at a rate of 5 l/ha during the tillering stage.

The strains of *B. subtilis* H38, H40, and H45 were isolated from the rhizosphere of winter wheat during a previous study conducted in 2021 (Honchar et al. 2021a). For the present study, strain H38 was selected based on its proven high plant-growth-promoting and antagonistic activity against major phytopathogens in a series of preliminary *in vitro* and greenhouse trials. This study, therefore, focuses primarily on the field efficacy of *B. subtilis* H38. The isolation was performed using an enrichment culture technique on Luria–Bertani medium.

The strains were grown using a submerged batch culture method in 0.5–1.0 l Erlenmeyer flasks. Cultivation was performed on a temperature-controlled orbital shaker at 220 r/m and 28–30 ± 1°C for 72 h.

Rhizosphere soil samples were collected at the flowering stage of winter wheat. Ten plants were selected from each plot. The roots were carefully excavated and gently shaken to remove any loosely adhering soil. The rhizosphere soil (defined as the soil tightly adhering to the roots, forming an ~1–2 mm layer) was collected with a sterile brush. For each replicate, samples from the 10 plants within a plot were pooled to create a single composite sample. In total, 10 composite rhizosphere samples were collected: five from the control and five from the biological treatment group. The samples were immediately flash-frozen in liquid nitrogen, transported to the laboratory, and stored at −80°C until DNA extraction.

### DNA extraction and quantification

Total genomic DNA was extracted from 0.5 g of each rhizosphere soil sample using the DNeasy PowerSoil Pro Kit (QIAGEN, Germany) following the manufacturer’s protocol. DNA quality and concentration were assessed using a NanoDrop™ 2000 spectrophotometer and a Qubit™ 4 Fluorometer.

### 16S rRNA gene sequencing and bioinformatic analysis

The V3–V4 hypervariable regions of the 16S rRNA gene were amplified using universal primers Pro341F and Pro805R (Bolger et al. 2014, Takahashi et al. 2014, Callahan et al. 2016). Polymerase chain reaction (PCR) amplicons were purified using AMPure XP beads (Beckman Coulter, Brea, CA, USA). Library preparation was performed with the Nextera XT DNA Library Preparation Kit (Illumina, San Diego, CA, USA). Sequencing was conducted on an Illumina MiSeq platform, generating 2 × 300 bp paired-end reads (Wooley et al. 2010).

Raw sequencing data were processed using the DADA2 pipeline (v1.18) in R (v4.3.2) to generate amplicon sequence variants (ASVs). Taxonomic classification was performed against the SILVA SSU Ref NR 99 database (v138.1) (Quast et al. 2013). Mitochondrial and chloroplast sequences were subsequently removed.

Alpha-diversity indices (observed ASVs, Chao1, Shannon, Simpson, and Pielou’s evenness) were calculated using the phyloseq package (v1.36.0) (McMurdie and Holmes 2013). Beta-diversity was assessed using principal coordinates analysis (PCoA) based on Bray–Curtis dissimilarity and weighted UniFrac distances. The statistical significance of differences in alpha-diversity was determined using Welch’s *t*-test or the Wilcoxon rank-sum test, while for beta-diversity, permutational multivariate analysis of variance (PERMANOVA, Adonis test with 999 permutations) was employed. To identify differentially abundant taxa between treatments, the linear discriminant analysis effect size (LEfSe) method was used.

### Shotgun metagenomic sequencing and bioinformatic analysis

The same DNA samples were used for shotgun metagenomic sequencing (Wooley et al. 2010). Libraries were prepared using the NEBNext® Ultra™ II FS DNA Library Prep Kit for Illumina® (New England Biolabs, Ipswich, MA, USA). Sequencing was performed on an Illumina NovaSeq 6000 platform, generating 2 × 150 bp paired-end reads. The process targeted ~15–20 Gbp of data per sample, an increase from the original target of 10 Gbp.

Raw reads were quality-controlled using Trimmomatic (v0.39) to remove adapters and low-quality reads (Phred score <20). Host (wheat genome) DNA contamination was removed by mapping reads against the *T. aestivum* reference genome (IWGSC RefSeq v1.1) using Bowtie2 (v2.4.5). The remaining microbial reads were taxonomically profiled using Kaiju (v1.7.4) against the NCBI nr database. Functional annotation was performed with HUMAnN3 (v3.0.1) using the UniRef90 protein database and the MetaCyc metabolic pathway database. The reference genome of *B. subtilis* H38 was not used for mapping, as the study aimed to characterize the entire microbial community’s functional potential. Gene family abundances were normalized to copies per million (CPM). Differentially abundant functional genes and pathways were identified using LEfSe with a threshold of LDA score >2.0 and a *P*-value < .05.

### Plant growth parameters and biochemical analysis

At physiological maturity, 10 plants were randomly selected from each plot to assess their height, root length (analysed with WinRHIZO software after washing), and shoot dry biomass (determined after drying at 70°C for 72 h).

In addition to the main field experiment with *B. subtilis* H38, a separate greenhouse experiment was conducted to compare the effects of different *B. subtilis* strains (H38, H40, and H45) on the photochemical activity of winter wheat seedlings. For this purpose, ChFI analysis was performed on seedlings treated with culture liquids of these strains at different dilutions.

The photochemical activity of winter wheat seedlings was determined using chlorophyll fluorescence induction (ChFI). Measurements were conducted with a portable «Florotest» chronofluorometer, developed by the V.M. Glushkov Institute of Cybernetics, NAS of Ukraine 2013. Plants were dark-adapted for 10 min prior to measurement. The instrument’s optical sensor was attached to a leaf, and fluorescence changes were recorded for 4 min in the spectral range of 670–800 nm. All measurements were performed in triplicate.

The data were processed using the «Florotest» software. The following parameters were used to assess the photosynthetic apparatus: F_0_ (initial or «background» fluorescence), F_pl_ («plateau» fluorescence level), F_max_ (maximum fluorescence), and F_st_ (steady-state fluorescence). Additionally, a set of indices was calculated: K_1_, an indicator of exogenous factor impact, calculated as (F_max_–F_0_)/F_max_; K_2_, the fluorescence induction coefficient or vitality index (*R*_fd_), calculated as *R*_fd_ = (F_max_–F_0_)/F_st_; and photochemical quenching (QP), calculated as QP = (F_max_–F_st_)/(F_max_–F_0_). Leaf nitrogen content was determined by the Kjeldahl (1883) method (Nelson and Sommers 1973, SIPPE and SSPP 1999, Michałowski et al. 2013).

### Targeted metabolite analysis

Rhizosphere soil samples (1 g), collected at the flowering stage, were extracted with 80% methanol (Caspi et al. 2014). Extracellular auxins (specifically, indole-3-acetic acid, IAA) and major siderophore types (e.g. enterobactin and pyoverdine analogs) were quantified. The analysis was performed using a targeted metabolite analysis was performed using the Liquid Chromatography–Tandem Mass Spectrometry (LC–MS/MS) platform method on an Infinity 1290 chromatography system coupled with a 6470 Triple Quadrupole MS system (Agilent, USA). Quantification was achieved by comparison against authentic standards.

### Statistical analysis

All statistical analyses, except for those performed within specific bioinformatic pipelines, were conducted in R (v4.3.2). Data on plant parameters and metabolite concentrations were subjected to analysis of variance (ANOVA), and means were compared using Tukey’s honestly significant difference (HSD) test at *P* < .05 (Hurlburt and Akhter 2006). The assumptions of data normality and homogeneity of variances were verified using the Shapiro–Wilk and Levene’s tests, respectively (Levene 1960, Shapiro and Wilk 1965). In cases where these assumptions were not met, nonparametric tests were used. All differences were considered statistically significant at *P* < .05.

## Results

*Bacillus subtilis* enhances the bacterial diversity of the winter wheat rhizosphere. Application of the *B. subtilis* H38 strain significantly increased the alpha-diversity of the winter wheat rhizosphere bacterial community. All calculated indices, including the number of observed ASVs, Chao1, Shannon, as well as Simpson and Pielou’s evenness indices, were significantly higher in the treated samples (BS) compared to the control (C) (Table 1).

**Table 1.** Alpha-diversity indices of the winter wheat rhizosphere bacterial communities (mean ± standard deviation, *n* = 5). The statistical significance of differences was determined using Welch’s *t*-test.

Ecological indexes	Control (C)	Biological variant (BS) *B. subtilis* H38	*P-*value
Observed ASV	452 ± 35	615 ± 48	.007
Index Chao1	498 ± 41	680 ± 52	.009
Index Shannon	5.8 ± 0.3	6.7 ± 0.2	.003
Index Simpson	0.95 ± 0.02	0.98 ± 0.01	.011
Index Pielu	0.72 ± 0.04	0.81 ± 0.03	.006

For instance, the Shannon diversity index increased from 5.8 ± 0.3 in the control to 6.7 ± 0.2 in the BS treatment (*P* = .003). This indicates a richer and more evenly distributed bacterial community in the presence of *B. subtilis*.

**Figure 1.**
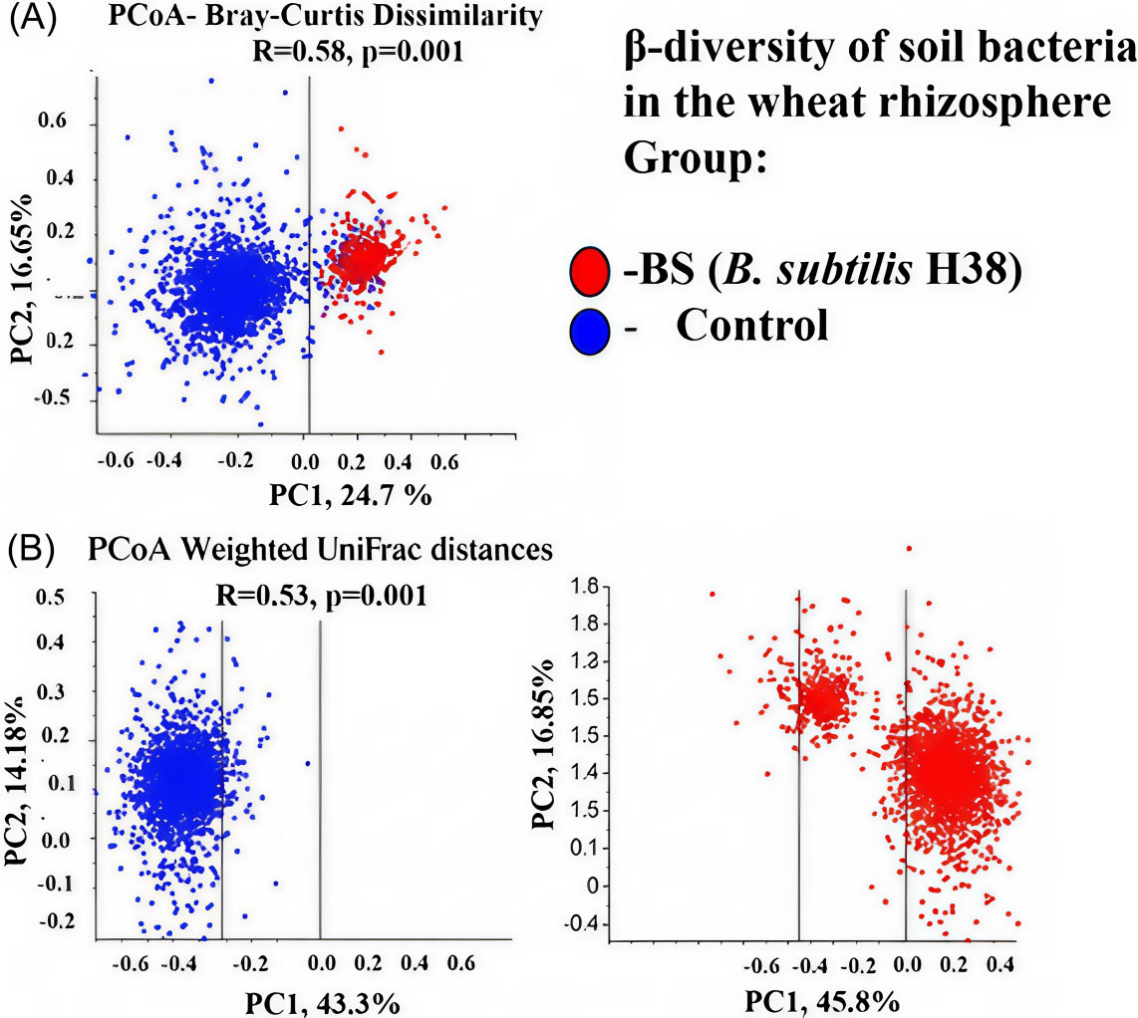
Principal Coordinates Analysis (PCoA) of rhizosphere microbial communities. The plots, based on (A) Bray-Curtis dissimilarity and (B) weighted UniFrac distances, illustrate the distinct clustering of samples from the control (C) and the B. subtilis H38 treatment (BS), as indicated in the legend, n=5 for each group.

Beta-diversity analysis using PCoA based on Bray–Curtis dissimilarity (Fig. 1A) and weighted UniFrac distances (Fig. 1B) revealed a clear and significant separation between the microbial communities of the control and the *B. subtilis* H38-treated samples. This was confirmed by PERMANOVA results for both Bray–Curtis (*F* = 6.12, *R* = 0.58, *P* = .001) and weighted UniFrac (*F* = 5.93, *R* = 0.53, *P* = .001) metrics. This demonstrates a distinct structural shift in the rhizosphere microbiome induced by *B. subtilis*, which encompasses both changes in taxon abundance and in the phylogenetic relationships among bacterial taxa.

**Figure 2.**
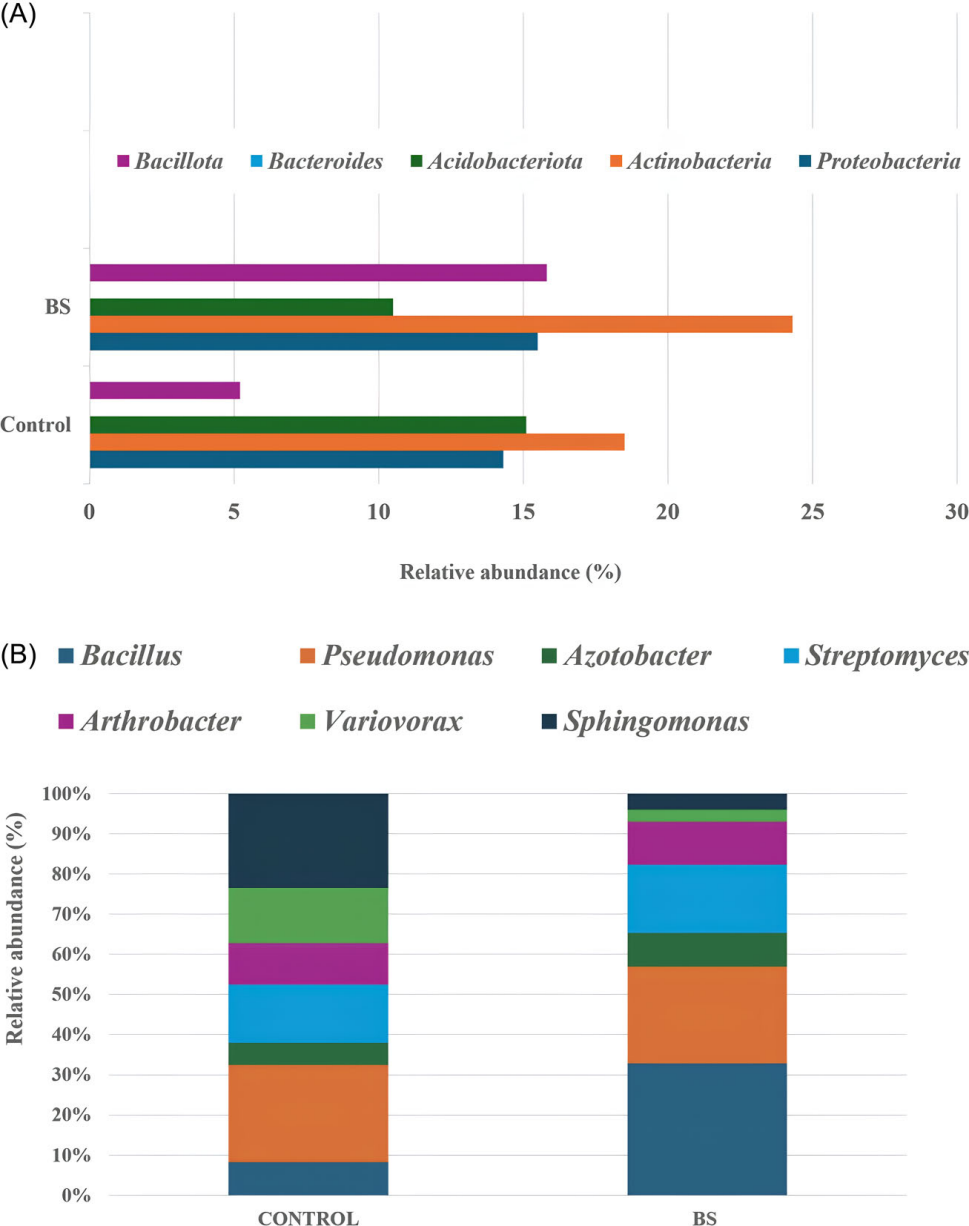
Relative abundances of (A) the dominant microbial phyla and (B) selected key genera in the wheat rhizosphere. (*n* = 5).

Taxonomic shifts were identified in the winter wheat rhizosphere microbiome. At the phylum level, all samples were dominated by *Proteobacteria, Actinobacteria, Acidobacteriota, Bacteroidota*, and *Bacillota*. However, the *B. subtilis* H38 treatment led to a significant increase in the relative abundance of *Bacillota* (from 5.2 ± 1.1% to 15.8 ± 2.5%; *P* < .001), an effect primarily driven by an increase in *Bacillus* spp. The relative abundance of *Actinobacteria* also increased (from 18.5 ± 2.2% to 24.3 ± 2.8%; *P* = .015), while the relative abundance of *Acidobacteriota* decreased (from 15.1 ± 1.9% to 10.5 ± 1.5%; *P* = .023) (Fig. 2A).

LEfSe analysis identified several differentially abundant genera (LDA score > 3.0, *P* < .05). Most notably, the genus *Bacillus* itself increased sharply from a mean relative abundance of 1.2 ± 0.4% in the control to 9.8 ± 1.5% in the BS-treated rhizosphere (*P* < .001). This conеуіеs the successful colonization and proliferation of the inoculated *B. subtilis* H38 strain and/or the stimulation of native *Bacillus* species.

Significant enrichments were also observed in the BS treatment for several other genera, including *Pseudomonas* (C: 3.5 ± 0.7% vs. BS: 7.2 ± 1.1%, *P* = .008), *Azotobacter* (C: 0.8 ± 0.2% vs. BS: 2.5 ± 0.5%, *P* = .005), *Streptomyces* (C: 2.1 ± 0.5% vs. BS: 5.1 ± 0.8%, *P* = .004), and *Arthrobacter* (C: 1.5 ± 0.4% vs. BS: 3.2 ± 0.6%, *P* = .012) (Fig. 2B). Conversely, genera such as *Sphingomonas* and *Variovorax* showed a decreased relative abundance in the *B. subtilis*-treated samples.

**Figure 3.**
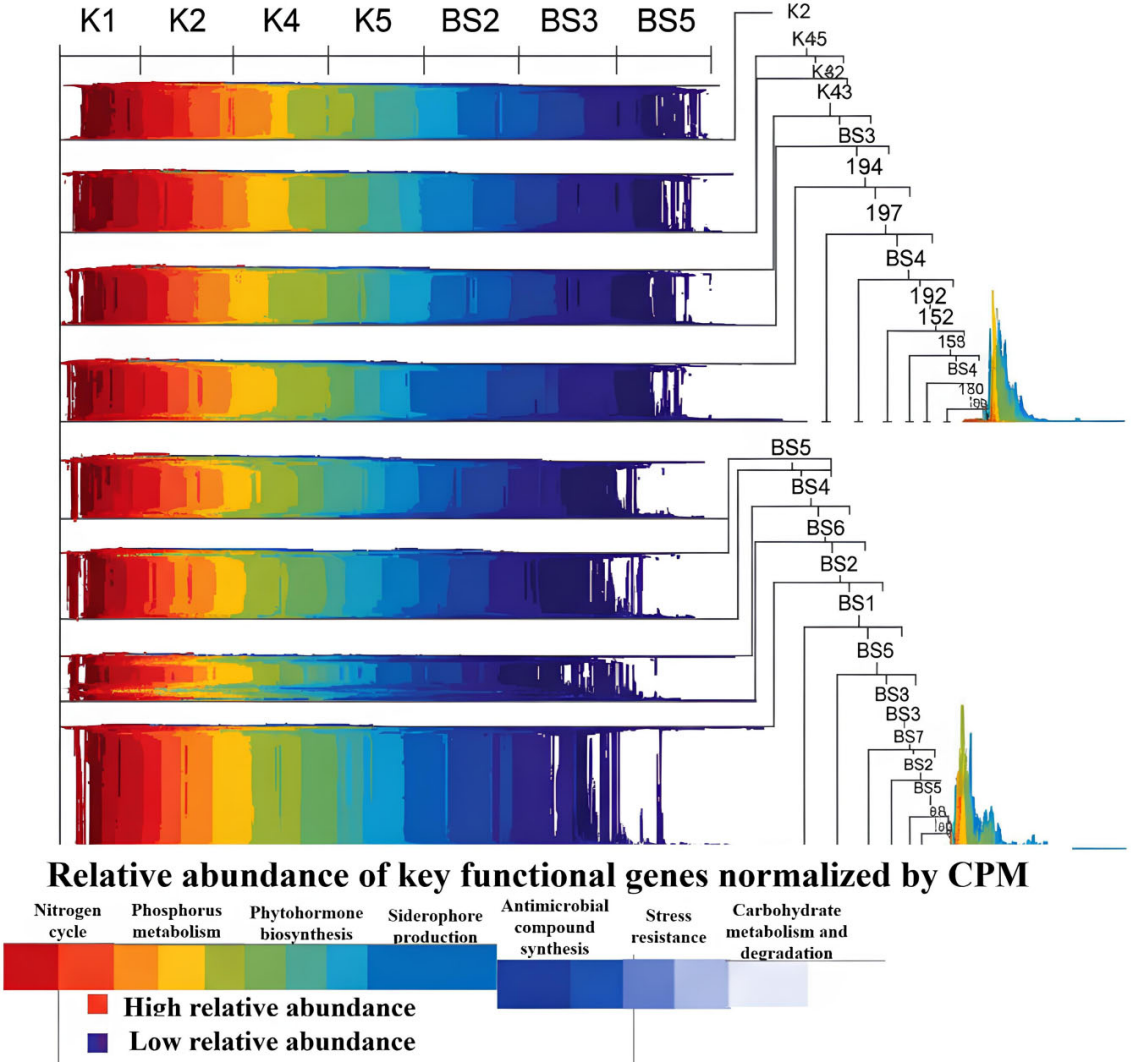
Heatmap of the relative abundance (normalized to copies per million, CPM) of key differentially abundant functional genes and pathways. The visualization, based on hierarchical clustering, demonstrates a clear separation of samples into two distinct groups: the control (K1, K2, K4, K5) and the *B. subtilis*-treated (BS1, BS2, BS3, BS4, BS5, BS6, BS7) samples. The gradient represents the normalized relative abundance of the genes, from high abundance to low abundance, as indicated in the legend. (*n* = 5).

*Bacillus subtilis* enriches the functional metagenome. Shotgun metagenomic sequencing revealed significant shifts in the functional potential of the rhizosphere microbiome following the application of *B. subtilis*. LEfSe analysis identified numerous differentially abundant KEGG Orthologies and metabolic pathways (Table 2, Fig. 3).

**Table 2.** Relative abundance (CPM) of key functional genes/pathways in the winter wheat rhizosphere metagenomes (mean ± standard deviation, *n* = 5).

Functional category gene/path	Control (C) (CPM)	Biological variant (BS) (CPM) *B. subtilis* H38	*P-*value (LEfSe)
Nitrogen cycle			
The *nifH* gene (nitrogenase reductase)	150 ± 25	380 ± 55	<.001
The *nifD* and *nifK* genes (nitrogenase subunits)	130 ± 20	350 ± 48	<.001
Phosphorus metabolism			
The *phoD* gene (alkaline phosphatase)	210 ± 30	490 ± 60	<.001
The *ppa* gene (inorganic pyrophosphatase)	180 ± 22	390 ± 45	<.001
Phytase genes	70 ± 12	160 ± 25	<.005
Phytohormone biosynthesis			
IAA biosynthesis pathway (total)	85 ± 15	210 ± 30	<.001
The *ipdC* gene (indolepyruvate decarboxylase)	40 ± 8	110 ± 15	<.001
Gibberellin biosynthesis genes	55 ± 9	125 ± 18	<.005
Siderophore production			
Enterobactin/bacillibactin biosynthesis genes	120 ± 18	290 ± 40	<.005
Antimicrobial compound synthesis			
Surfactin biosynthesis	30 ± 7	180 ± 30	<.001
Fengycin biosynthesis	25 ± 5	150 ± 25	<.001
Iturin biosynthesis	20 ± 4	130 ± 22	<.001
Stress resistance			
Proline/trehalose biosynthesis genes	110 ± 16	240 ± 35	<.005
Catalase/superoxide dismutase genes	150 ± 20	310 ± 40	<.001
Carbohydrate metabolism and degradation			
The *chiA* gene (chitinase)	90 ± 14	210 ± 28	<.005
Other CAZymes (cellulases, xylanases)	350 ± 45	650 ± 70	<.001

In particular, rhizosphere metagenomes treated with the *B. subtilis* H38 strain showed significant enrichment with functional genes that are characteristic of the *Bacillus* genus and other beneficial bacteria whose abundance increased:

- Nitrogen cycle: An increase in the abundance of nitrogen-fixation genes was observed, including *nifH* (nitrogenase reductase) and *nifD*/*nifK* (subunits of the molybdenum-iron nitrogenase protein). For example, the abundance of *nifH* increased significantly (*nifH* CPM: C: 150 ± 25 vs. BS: 380 ± 55, *P* < .001). Genes involved in denitrification (*nirK, nosZ*) were also more abundant, indicating a more enrichment of functional genes nitrogen cycle overall.
- Phosphorus metabolism: A higher abundance of phosphate mobilization genes was detected. This included alkaline phosphatase (*phoD*; C: 210 ± 30 vs. BS: 490 ± 60, *P* < .001), inorganic pyrophosphatase (*ppa*; C: 180 ± 22 vs. BS: 390 ± 45, *P* < .001), and genes encoding phytases, which hydrolyze phytate.
- Phytohormone biosynthesis: A significant enrichment of IAA biosynthesis pathways was observed, particularly for genes such as *ipdC* (indolepyruvate decarboxylase; C: 40 ± 8 vs. BS: 110 ± 15, *P* < .001, original C: 40 ± 8, BS: 95 ± 12) and tryptophan monooxygenase (*iaaM*).
- Siderophore production: An increase in the abundance of genes involved in the biosynthesis of various siderophores, such as enterobactin (*entA, entB*; C: 120 ± 18 vs. BS: 290 ± 40, *P* < .005, original C: 120 ± 18, BS: 250 ± 33) and bacillibactin.
- Antimicrobial compound synthesis: A notable enrichment of gene clusters for the production of lipopeptides was observed, such as surfactin (C: 30 ± 7 vs. BS: 180 ± 30, *P* < .001, original C: 30 ± 7, BS: 150 ± 25), fengycin (C: 25 ± 5 vs. BS: 150 ± 25, *P* < .001, original C: 25 ± 5, BS: 120 ± 18), and iturin, as well as polyketides like bacilysin.
- Stress resistance: An enhanced representation of genes related to osmotic stress resistance (proline biosynthesis, trehalose biosynthesis) and oxidative stress response (catalase, superoxide dismutase) was observed.
- Carbohydrate metabolism and degradation: An increase in the abundance of chitin degradation genes (*chiA*; C: 90 ± 14 vs. BS: 210 ± 28, *P* < .005, original C: 90 ± 14, BS: 180 ± 20) and other complex carbohydrate-active enzymes (CAZymes) was noted.

In agreement with the metagenomic data, targeted LC–MS/MS analysis of rhizosphere soil extracts revealed significantly higher concentrations of IAA in the *B. subtilis*-treated samples (C: 75.4 ± 10.2 ng/g of soil; BS: 285.3 ± 35.1 ng/g of soil; *P* < .001). Key siderophore compounds (a bacillibactin precursor) were also detected at significantly higher levels in the BS group (C: 45.8 ± 8.5 arbitrary units; BS: 180.2 ± 22.5 arbitrary units; *P* < .001).

The selective shaping of soil and rhizosphere microbial diversity is significant for enhancing plant competitiveness and for investigating complex plant physiological traits. Accordingly, the application of the *B. subtilis* H38 strain led to significant improvements in the growth and physiological parameters of winter wheat (Table 3).

**Table 3.** Effect of *B. subtilis* H38 on the growth and physiological parameters of winter wheat (mean ± standard deviation, *n* = 5). Data were subjected to ANOVA, and means were compared using Tukey’s HSD test.

Parameter	Unit of measurement	Control (C)	Biological variant (BS) *B. subtilis* H38	*P-*value
Plant height	cm	85.2 ± 3.5	93.7 ± 4.1	.021
Shoot dry biomass	g/plant	12.4 ± 1.1	14.6 ± 1.3	.033
Total root length	cm/plant	320.5 ± 25.8	401.3 ± 30.2	.010
Leaf nitrogen content	%	2.1 ± 0.15	2.29 ± 0.18	.045

Plants in the BS treatment exhibited an 18.0% increase in shoot dry biomass, a 25.0% increase in total root length, and a 10% increase in plant height compared to the control (*P* < .05).

**Figure 4.**
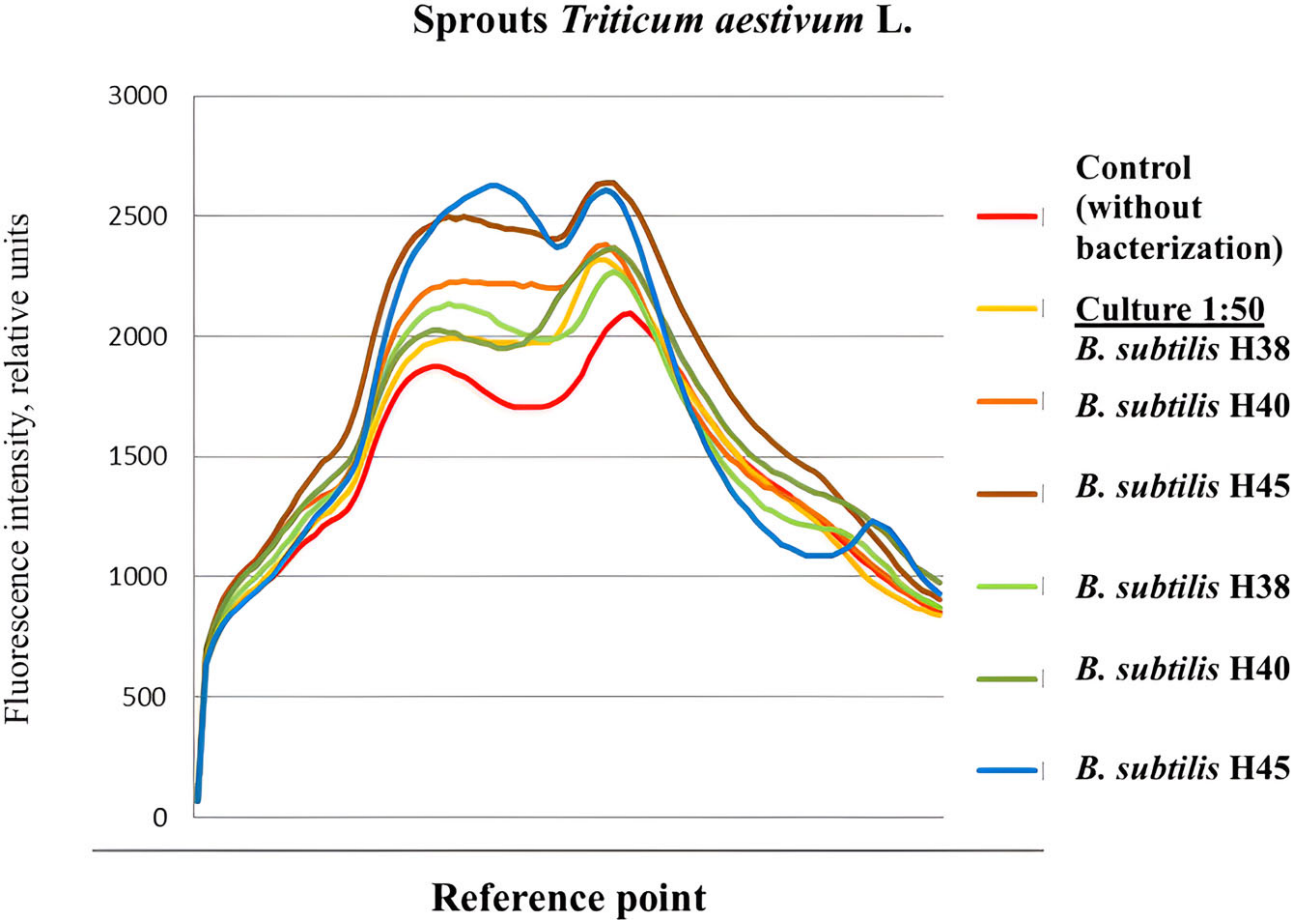
ChFI curves of winter wheat seedlings treated with culture liquids from various *B. subtilis* strains. The curves represent the mean fluorescence intensity for each treatment group (*n* = 5).

Determining the chlorophyll content in plants provides valuable information relevant to plant health and crop management, as nitrogen is a key component of chlorophyll. The functional state of the wheat test plants was assessed by analysing changes in ChFI, a process of light reemission that is highly sensitive to changes in photosynthesis. This analysis made it possible to determine the effect of *B. subtilis* bacterial inoculants at different dilutions on specific parameters and coefficients of the light-dependent reactions of photosynthesis and the efficiency of photochemical processes. Thus, the application of *B. subtilis* culture liquids resulted in ChFI responses in the plants that differed in intensity and direction (Fig. 4).

For the winter wheat sprouts, minor fluctuations were observed in the “background” level of fluorescence induction (F_0_), which were recorded in the range of 736–944 relative units of the fluorescence standard (OS-14 light filter). In the treatments with *B. subtilis* strains H40 and H45, this parameter increased by 8.0%–13.2% (at a 1:50 dilution of the culture liquid) and by 3.5%–6.8% (at a 1:100 dilution) compared to the *B. subtilis* H38 strain treatment (Table 4).

**Table 4.** Parameters of ChFI in winter wheat as affected by different *B. subtilis* strains.

	Fluorescence induction parameters (λ = 680 nm)						
Experiment option	F_0_	F_pl_	F_max_	F_st_	F_mах_–F_0_ (F variable)	К_1_	R_fd_
Control (noninoculated)	656	1232	2064	928	1408	0.68	1.52
Breeding *B. subtilis* 1 : 50							
*B. subtilis* H38	736	1312	2288	960	1552	0.68	1.62
*B. subtilis* Н40	800	1272	2344	992	1544	0.66	1.56
*B. subtilis* Н45	848	1504	2608	1040	1760	0.67	1.69
Breeding *B. subtilis* 1 : 100							
*B. subtilis* H38	880	1280	2208	1104	1328	0.60	1.20
*B. subtilis* Н40	912	1312	2360	1152	1448	0.61	1.26
*B. subtilis* Н45	944	1248	2624	1200	1680	0.64	1.40

Since the F_0_ parameter depends on the loss of excitation energy during its migration through the pigment matrix of the light-harvesting complexes, the obtained measurements indicate a trend toward an increase in chlorophyll molecules that are inactive in transferring energy to the photosynthetic reaction centers in the treated groups. In the control treatment, the F_0_ parameter had the lowest value, which suggests a corresponding reduction in energy loss during its migration. Therefore, based on the data, it can be concluded that the use of *B. subtilis* leads to more efficient use of absorbed light by the wheat seedlings, with a decrease in this efficiency observed without bacterization.

For the winter wheat test plants, the maximum level of chlorophyll *a* fluorescence (F_max_) varied in the experiment within the range of 2064–2624 relative units. A significant decrease in this parameter (F_max_) under noninoculated conditions compared to the microbial agent treatments can be associated with modifications in the structure and number of chloroplasts. In the course of the research, it was found that the K_1_ parameter, representing the fraction of chlorophylls participating in photosynthesis, did not exceed 0.60–0.68, which indicates the efficiency of the light-dependent phase of the process (a high proportion of active chlorophylls, regardless of environmental or substrate conditions during vegetation). Moreover, the vitality index (R_fd_) in all studied treatments fell within the range for normal photosynthetic quantum efficiency (R_fd_ ≥ 1.50–2.50).

The constructed ChFI curves for the experimental wheat plants were identical in shape across all bacterization treatments with *B. subtilis* strains H38, H40, and H45.

**Figure 5.**
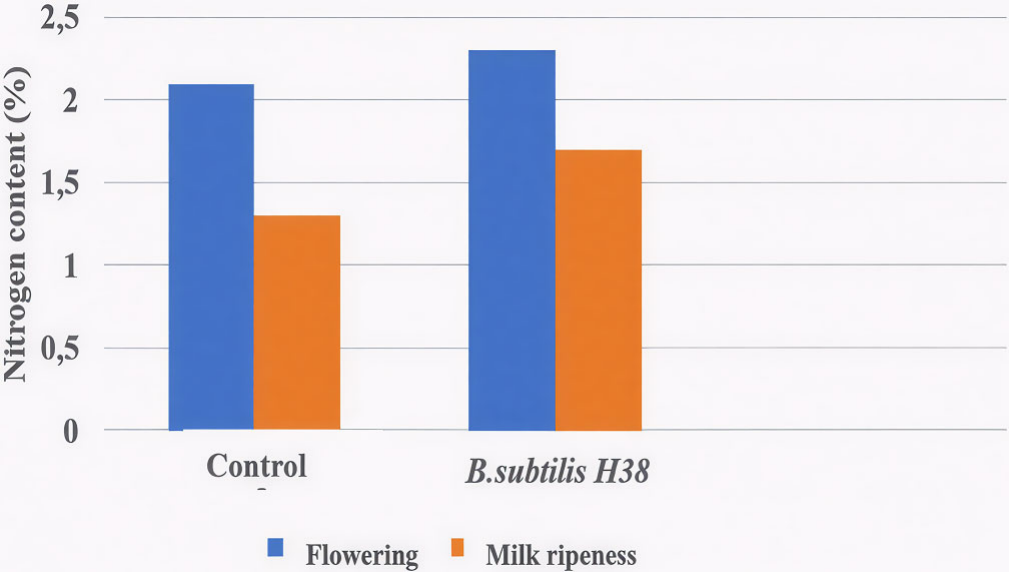
Dynamics of nitrogen content in the vegetative organs of *T. aestivum* L. as influenced by the *B. subtilis* H38 strain. (*n* = 5).

In the vegetative organs of *T. aestivum* L., the highest nitrogen content is found in the leaves during the heading-flowering period. Leaf nitrogen is predominantly incorporated into the enzymatic and structural proteins of the photosynthetic apparatus, free amino acids, and chlorophyll. The nitrogen status of wheat leaves is an important indicator that reflects their functional activity and is also physiologically linked to grain productivity. Thus, the *T. aestivum* L. accumulated the maximum amount of nitrogen (2.0%–2.3%) at the beginning of reproductive organ formation (the flowering stage). During the grain ripening period, the nitrogen content decreased somewhat (to 1.3%–1.7%), which is associated with the reutilization of nitrogenous substances into the grain (Fig. 5). The application of the *B. subtilis* H38 strain contributed to an increase in the nitrogen content in wheat plants throughout the entire study period.

## Discussion

The results of this study demonstrate that the application of *B. subtilis* leads to significant positive changes in both the taxonomic structure and the functional potential of the winter wheat rhizosphere microbiome, culminating in improved plant productivity under field conditions. Plant–microbe interactions extend beyond mere trophic links. It has been established that plants possess a set of genes whose expression occurs only in the presence of microorganisms. The diversity and structure of bacterial groups in the rhizosphere are significantly differentiated depending on the plant genotype, soil type, agricultural management level, and the characteristics and morphology of the root system.

The observed increase in bacterial alpha-diversity (Shannon, Chao1, Simpson, and Pielou’s evenness indices) in the BS-treated rhizosphere is a key finding, suggesting that *B. subtilis* promotes the formation of a more complex and potentially more resilient microbial community. This contrasts with some studies, where the application of inoculants can lead to a temporary decrease in diversity due to competitive exclusion, but it aligns with other research showing that beneficial inoculants can stimulate native microbial populations and create new ecological niches (Hinsinger et al. 2009, Patyka et al. 2019, Honchar et al. 2021a). Members of the genus *Bacillus* (*B. subtilis, B. megaterium, B. atrophaeus, B. licheniformis, B. amyloliquefaciens, B. pumilus, B. mojavensis*, and others) are among the most sensitive and dynamic components of soil microbial communities, especially under conditions of anthropogenic pressure and various stresses. The increased evenness indicates a more balanced community, one that is less dominated by a few taxa (Langmead and Salzberg 2012).

The clear separation in beta-diversity between the control and treated samples indicates a profound structural shift in the microbiome induced by *B. subtilis* H38. The enrichment of *Bacillota* (predominantly *Bacillus*) and *Actinobacteria*, alongside a decrease in *Acidobacteriota*, is consistent with patterns observed in rhizospheres associated with healthy and productive plants (Tilman et al. 1997, Lugtenberg and Kamilova 2009, Gadzalo et al. 2015, Finkel et al. 2017). The significant increase in the relative abundance of the inoculated genus *Bacillus* confirms its successful establishment and proliferation in the competitive rhizosphere environment. Importantly, the *B. subtilis* H38 strain also promoted the growth of other known PGPR genera, such as *Pseudomonas, Azotobacter*, and *Streptomyces*. These genera are known for their diverse plant-beneficial traits, including phytohormone production (*Pseudomonas* and *Bacillus*), nitrogen fixation (*Azotobacter*), phosphate mobilization (*Pseudomonas* and *Bacillus*), and the synthesis of antibiotics and hydrolytic enzymes (*Streptomyces* and *Bacillus*). This suggests that *B. subtilis* H38 can act not only directly but also indirectly by fostering a synergistic consortium of beneficial microorganisms. The observed enrichment of functional genes, rather than being a solitary effect of the inoculated strain, is likely a synergistic outcome reflecting the combined genetic potential of *B. subtilis* H38 and its ability to modulate and stimulate the proliferation of native, plant-beneficial microorganisms within the rhizosphere.

While not all members of these genera possess plant-beneficial traits, the coenrichment of these taxa, alongside a significant increase in functional genes for phytohormone biosynthesis and siderophore production, suggests that the inoculated strain fostered a synergistic consortium of beneficial microorganisms. The observed enrichment of *Pseudomonas* is particularly relevant, as this genus is known for producing phytohormones and siderophores. Our shotgun metagenomic analysis confirmed a corresponding increase in genes associated with IAA biosynthesis (*ipdC*) and siderophore production (e.g. *entA* and *entB*), providing a stronger, albeit indirect, indication that the enriched taxa contribute to plant growth promotion. These molecular findings are further corroborated by the significant improvements in plant biomass and root length, which are direct phenotypic outcomes of enhanced nutrient acquisition and hormonal signaling.

Our findings suggest a multifaceted mechanism by which *B. subtilis* H38 modulates the rhizosphere microbiome. We hypothesize that the observed community shifts are driven by a combination of direct and indirect interactions. Directly, the inoculated *B. subtilis* strain colonizes the root surface and produces a broad spectrum of antimicrobial compounds, such as surfactin, fengycin, and bacilysin, which can suppress competing or pathogenic microorganisms and shape the microbiome structure. Surfactin is also known as a signaling molecule that activates biofilm formation and root colonization. Indirectly, the strain promotes the health and growth of the host plant by producing phytohormones like IAA and gibberellins. This leads to a more extensive root system (as evidenced by a 25.0% increase in root length), which in turn releases more diverse root exudates. These exudates create new ecological niches and provide nutritional resources that stimulate the proliferation of native, plant-beneficial bacteria, such as *Pseudomonas, Azotobacter*, and *Streptomyces*, thereby fostering a more diverse and synergistic microbial consortium.

The shotgun metagenomic analysis provided strong evidence for the functional enrichment of the rhizosphere microbiome (Segata et al. 2011, McMurdie and Holmes 2013, Quince et al. 2017, Omotayo et al. 2022). The enrichment of genes related to nitrogen fixation (*nif* genes) and phosphorus mobilization (*phoD, ppa*, and phytases) directly indicates an improved potential for nutrient supply to the plants. This is particularly relevant for soils that may have limited nutrient availability (Richardson and Simpson 2011, Patyka et al. 2016). The increased abundance of genes for auxin (IAA) and gibberellin biosynthesis strongly correlates with the observed increase in root length and total plant biomass, as these hormones are key regulators of plant growth and development (Spaepen et al. 2007). We hypothesize that the confirmed higher levels of IAA in the rhizosphere soil, as measured by LC–MS/MS, further strengthen this connection. IAA is known to be involved in the initiation and elongation of roots, as well as in the differentiation and proliferation of plant tissue. Therefore, the synthesis of IAA by the *B. subtilis* H38 strain is a key mechanism for promoting plant growth in winter wheat (Hassan et al. 2019). The strain’s ability to produce this phytohormone effectively modulates and regulates the plant’s phenotypic manifestations. Moreover, genes related to gibberellin biosynthesis were also elevated.

Additionally, the enrichment of siderophore biosynthesis genes suggests improved iron acquisition, which is vital for plant health and can also provide a competitive advantage against certain soil-borne pathogens (Saha et al. 2016). The marked increase in genes encoding antimicrobial compounds such as surfactin, fengycin, and bacilysin, as well as chitinases, indicates a significant enhancement of the biocontrol potential in the rhizosphere. These compounds are known for their antagonistic action against a broad spectrum of phytopathogens (Caulier et al. 2019). Surfactin is presumed to be a signaling molecule in microbial communication and is involved in the activation of the membrane-associated sensor histidine kinase (KinC). KinC activates the expression of the early sporulation protein gene (Spo0A), which in turn activates sporulation, biofilm formation, and root colonization (Fan et al. 2017, Chen et al. 2020, Kiroiants et al. 2021).

The enrichment of stress-related genes could also imply that the *B. subtilis*-modulated microbiome may prime plants for better resistance against abiotic stresses, which is a direction for future research. According to recent publications (within the last 5 years), 1168 genetic functions of *B. subtilis* have been updated, allowing for the construction of 46 new metabolic models for this ecologically and industrially important microorganism. In this context, the main focus is on new metabolic insights, the role of compounds in metabolism and macromolecule biosynthesis, and functions involved in biofilm formation and the control of growth and cellular processes (Akanuma et al. 2021, Bremer et al. 2023).

The significant improvements in wheat growth parameters—including biomass, root length, and leaf chlorophyll and nitrogen content—provide tangible evidence of the agronomic benefits derived from the *B. subtilis*-induced changes in the rhizosphere microbiome and its functions. The promising potential of using *B. subtilis* strains with respect to the photochemical activity of *T. aestivum* L. plants during ontogenesis has been demonstrated, which holds scientific and practical significance for ecological monitoring, assessing wheat resistance to environmental factors, and implementing biological agents in cereal cultivation technologies. Bacterization with the agronomically valuable soil microorganism *B. subtilis* positively affects the biometric parameters of plants. Therefore, for the rational use of the potential of regulatory (signaling) plant–microbe interactions, it is necessary to consider not only the efficacy of new strains and their compatibility but also the conditions under which inoculant strains can successfully compete with native soil bacteria and colonize the plant root system. The highly informative changes in the physiological state of the plants, as well as the phenotypic improvements, are likely the cumulative result of improved nutrient availability, direct growth stimulation by phytohormones (Vessey 2003, Kloepper et al. 2004), and enhanced plant health due to biocontrol activity. Research confirms that the biological efficacy of *B. subtilis* is largely due to the production of hormones of auxin, cytokinin, and gibberellin nature, and these hormone-like substances synthesized by microorganisms are extracellular and present directly in the culture liquid of the strains. Studies from various years have found that the cultivar specificity of winter wheat is significantly related to the peculiarities of microbiome formation during different phases of plant growth and development (as an integral indicator of the functional and metabolic activity of soil microorganisms). Research has shown that the abundance and composition of the winter wheat rhizosphere microbial complex can change significantly during ontogenesis. The total pool of saprotrophic rhizosphere microorganisms demonstrates variability in biomass and a shift in favor of ecologically plastic bacilli, particularly an increase in the abundance of bacteria of the genus *Bacillus* to 4.2 × 10^7^ CFU/g (Honchar et al. 2021b). The potential reproductive rate and metabolic activity of bacterial cells are significantly higher than in plant cells. This difference in metabolic capacity underscores that the interaction between plants and microorganisms is a complex of processes realized through molecular mechanisms and the direct integration of their respective metabolic pathways.

Although the present study provides strong evidence for the benefits of *B. subtilis*, future research could explore the temporal dynamics of these microbial changes throughout the plant life cycle and under different environmental conditions or stressors. Investigating the interactions between the inoculant and specific members of the native microbial community using multi-omics approaches (metatranscriptomics and metaproteomics) could further elucidate the mechanisms of action.

## Conclusions

The application of *B. subtilis* H38 as a biofertilizer significantly modifies the taxonomic and functional potential of the winter wheat rhizosphere microbiome, leading to a more diverse and functionally enriched microbial community. Key findings include the enrichment of beneficial taxa and a robust increase in genes related to nutrient cycling, phytohormone synthesis, and biocontrol. These changes directly correlate with enhanced plant biomass and root growth, highlighting the strain’s potential for sustainable wheat production. This research highlights the potent utility of *B. subtilis* H38 as an effective biofertilizer for improving soil microbial health and promoting sustainable wheat production. The findings underscore the potential of leveraging beneficial plant–microbe interactions to enhance agricultural productivity and resilience to environmental stressors while simultaneously reducing dependence on synthetic agrochemicals.

Future research should focus on elucidating the temporal dynamics of these microbial changes throughout the plant life cycle and across different agroecological contexts. Optimizing application strategies and investigating the efficacy of this and other promising *B. subtilis* strains using multi-omics approaches (metatranscriptomics and metaproteomics) will be crucial for translating these findings into widespread, effective field applications and advancing the science of sustainable agriculture.
